# Cockroach sensitization and its hidden links to mite and food allergens

**DOI:** 10.1038/s41598-026-44011-8

**Published:** 2026-03-11

**Authors:** Marharyta Sobczak, Patrycja Kitlas, Rafał Pawliczak, Krzysztof Kowal

**Affiliations:** 1 Department of Immunopathology, Division of Biomedical Science, Faculty of Medicine, Medical University of Lodz, Lodz, Poland; 2 Department of Experimental Allergology and Immunology, Department of Allergology and Internal Medicine, Medical University of Bialystok, Bialystok, Poland

**Keywords:** Cockroach sensitization, Cross-reactivity, Allergy, Allergy rhinitis, ALEX test, Skin prick test, Diseases, Ecology, Ecology, Zoology

## Abstract

Cockroach allergy is a common trigger of allergic reactions and may be a cause or a result of cross-reactions with other allergens. The aim of this study was to assess the pattern of sensitization to arthropod allergens in perennial allergic rhinitis (PAR) patients with positive skin prick test to cockroach. A group of PAR patients with positive skin prick test (SPT) result with cockroach extract (*Blattella germanica*) was selected. In addition to SPTs for other inhalant allergens, such as house dust mites *(Dermatophagoides pteronyssinus and Dermatophagoides farinae)*, birch, grass, mugwort, cat, dog, and *Alternaria*, participants underwent the ALEX2 test which allowed for detection of sensitization to cockroach-specific and cross-reacting molecules. Forty-eight participants took part in the study, of whom forty-six underwent the ALEX2 test. Among PAR patients with positive SPT results to cockroach extract only 2 had elevated IgE levels to cockroach specific allergens (Bla g 1 and Bla g 4). However, in substantial number of patients sensitization to cross-reacting allergens was demonstrated. This was associated with frequent sensitization to other arthropod extracts. A correlation was observed between cockroach allergy and allergy to edible insects such as crickets, locusts, and mealworms; seafood; house dust mites and storage mites; and wasp species, depending on the cockroach species. In our population of PAR patients sensitization to cockroaches is associated with a broader spectrum of cross-reactive allergens. These findings deepen our understanding of potential cross-allergenicity and may form the basis for personalized risk assessment and allergy treatment in patients with AR.

## Introduction

Cross-reactivity is defined as an adaptive immune response to one antigen that leads to reactivity with other antigens. This is due to the structural similarity of the allergen epitopes^1,2^. It is interesting to note that cross-reactivity between two proteins is likely when their sequences are more than 70% identical, whereas with a similarity of less than 50%, the possibility of such a reaction is low^1^. On the one hand, cross-reactivity can protect against infections, but on the other hand, it has a negative impact on immune disorders, including allergic diseases^2^. Of course, it is important to distinguish between cross-reactivity and co-sensitization to specific allergens. Skin and serological tests have been used for many years to diagnose inhalant allergies, and their results are compared with clinical history^3^. There are two types of tests for specific IgE: singleplex and multiplex. Singleplex tests allow the measurement of IgE antibody concentrations against a single allergen, while multiplex tests allow the simultaneous determination of IgE against multiple allergens. One of the multiplex systems is the ALEX macroarray, which has been available since 2016. Multiplex tests do not require prior selection of allergens and prove particularly useful in patients whose allergy diagnosis is difficult^4^.

According to the systematic nomenclature of allergens^5^, there are currently 33 cockroach allergens, divided into two species: German cockroach (*Blattella germanica*) and American cockroach (*Periplaneta americana*). Bla g 1 Bla g 2 and Bla g 4 are specific to cockroaches and useful for identifying a true cockroach allergy. In contrast, Bla g 5 Bla g 7 or Bla g 9 are cross-reactive allergens^6^. The prevalence of allergen-specific IgE among cockroach allergic patients is 20–50% for Bla g 1, 18–73% for Bla g 2, while among cross-reactive Bla g 5 and Bla g 7–39–73% and 16%, respectively. Interestingly, among in those allergic to *Periplaneta americana*, the prevalence rate of sensitization to Per a 5 is 100% and for Per a 7 is between 13% and 54%^7^. A cross-sectional study conducted in the Czech Republic revealed that sensitivity to at least one cockroach-specific molecule (such as Bla g 1, Bla g 2, or Bla g 5) occurred in only 0.6% of patients suspected of suffering from allergic diseases. Furthermore, nearly all of these cases demonstrated sensitization to molecules other than cockroach molecules^8^. Cross-reactivity has been demonstrated between different species of cockroaches, as well as between cockroaches and other insect, arachnid, and crustacean families^3^. However, sensitization to cockroaches is one of the risk factors for developing asthma^9^. In a population of Polish children with allergic asthma, sensitization to cockroach extract was the greatest in those with severe asthma^10^. In a population of 67 Thai patients with allergic rhinitis (AR), 32 tested positive for American cockroach allergen using both skin prick test and specific IgE (sIgE). Interestingly, 20.9% of cases had negative sIgE but positive skin prick test for the American cockroach, while 10.4% had negative skin prick test but positive sIgE^11^. However, those studies did not evaluate molecular patterns of sensitization to cockroaches.

Shellfish are one of the most frequent triggers of food allergies, and crustacean allergens may cross-react with those from cockroaches and house dust mites. Although analysis of amino acid sequence similarity and epitope reactivity has indicated that lobster tropomyosin has the highest level of cross-reactivity with shrimp allergens, and cockroach tropomyosin has the lowest, it is still highly cross-reactive allergen^12^. Therefore, the aim of our study was to evaluate molecular pattern of IgE reactivity to cockroach allergens among patients with perennial allergic rhinitis (PAR) with positive skin prick test to cockroach extract in the Polish population.

## Results

### Characteristics of participants

Forty-eight participants were enrolled in the study, of whom 46 underwent the ALEX2 test, as two patients did not consent to giving blood for in vitro testing. All participants had PAR and a positive SPT for cockroach extract (*Blattella germanica*). Characteristics of the study population are summarized in Table 1.

**Table 1:** Characteristics of the study population.

Characteristics	Total (*n* = 48)
Sex (F: M)	18:30
Age (y), mean (SD)	33.5 (12.7)
Perennial allergic rhinitis, N (%)	48 (100%)
Asthma, N (%)	18 (37.5%)
Atopic dermatitis, N (%)	10 (20.8%)
Urticaria, N (%)	2 (4.2%)

### Analysis of sensitization based on the results of skin prick tests

**Fig. 1:**
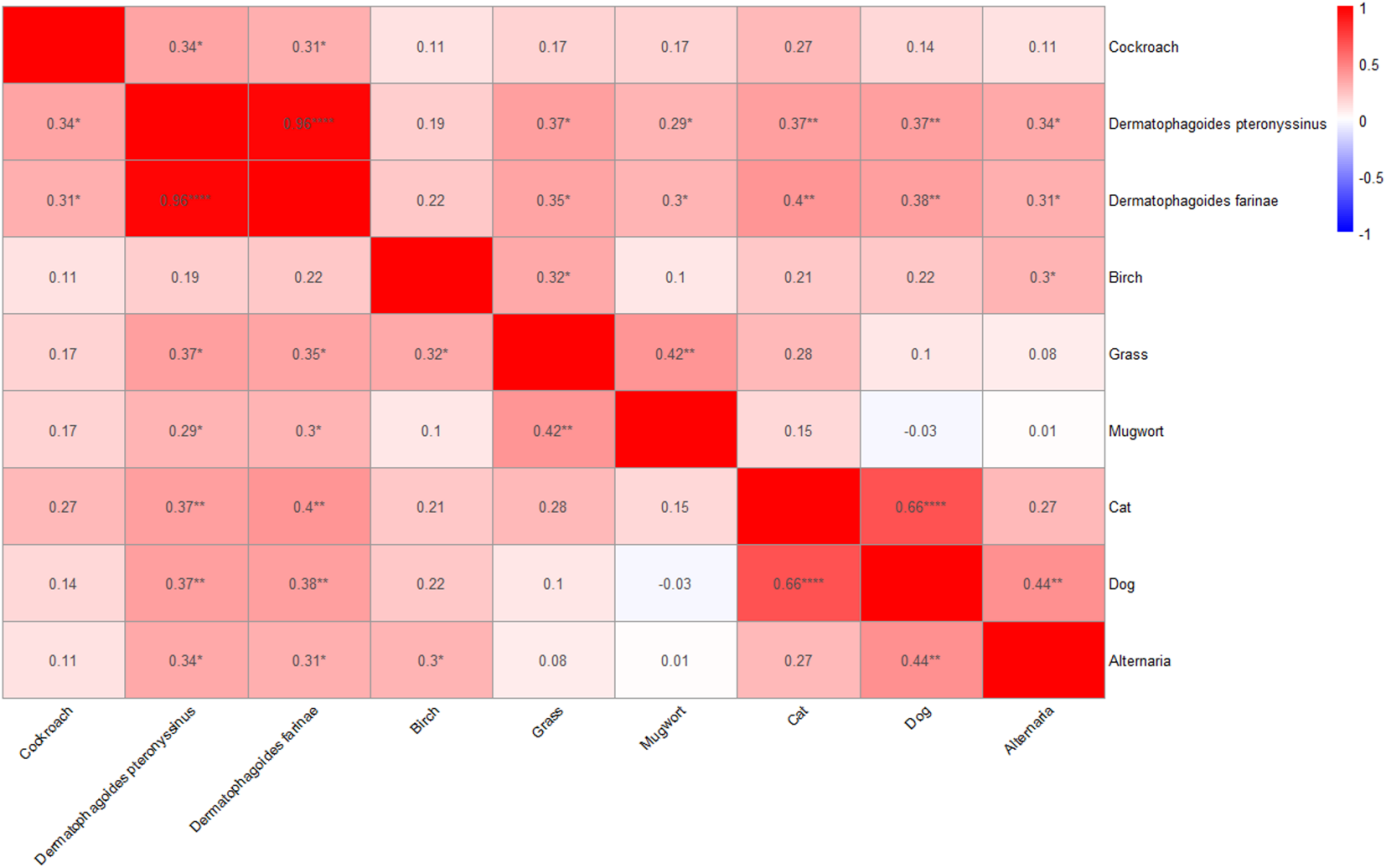
Correlation analysis of sensitization based on the results of skin prick tests. The values shown on the heat map are Spearman’s correlation coefficients. *p < 0.05, **p < 0.01, ****p < 0.0001.

In the first stage, we analyzed the correlation of skin prick test results for all 48 participants (Fig. 1). We detected a moderate positive correlation between cockroach allergen (*Blattella germanica*) and house dust mite allergens, both *Dermatophagoides pteronyssinus* (rho = 0.34, *p* = 0.02) and *Dermatophagoides farinae* (rho = 0.31, *p* = 0.03). In addition, our analysis shows a strong positive correlation between house dust allergens *Dermatophagoides pteronyssinus* and *Dermatophagoides farinae* (rho = 0.96, *p* < 0.0001), as well as between cat and dog allergens (rho = 0.66, *p* < 0.0001).

### Comparison of cockroach allergy test results based on skin prick tests and ALEX2

**Fig. 2:**
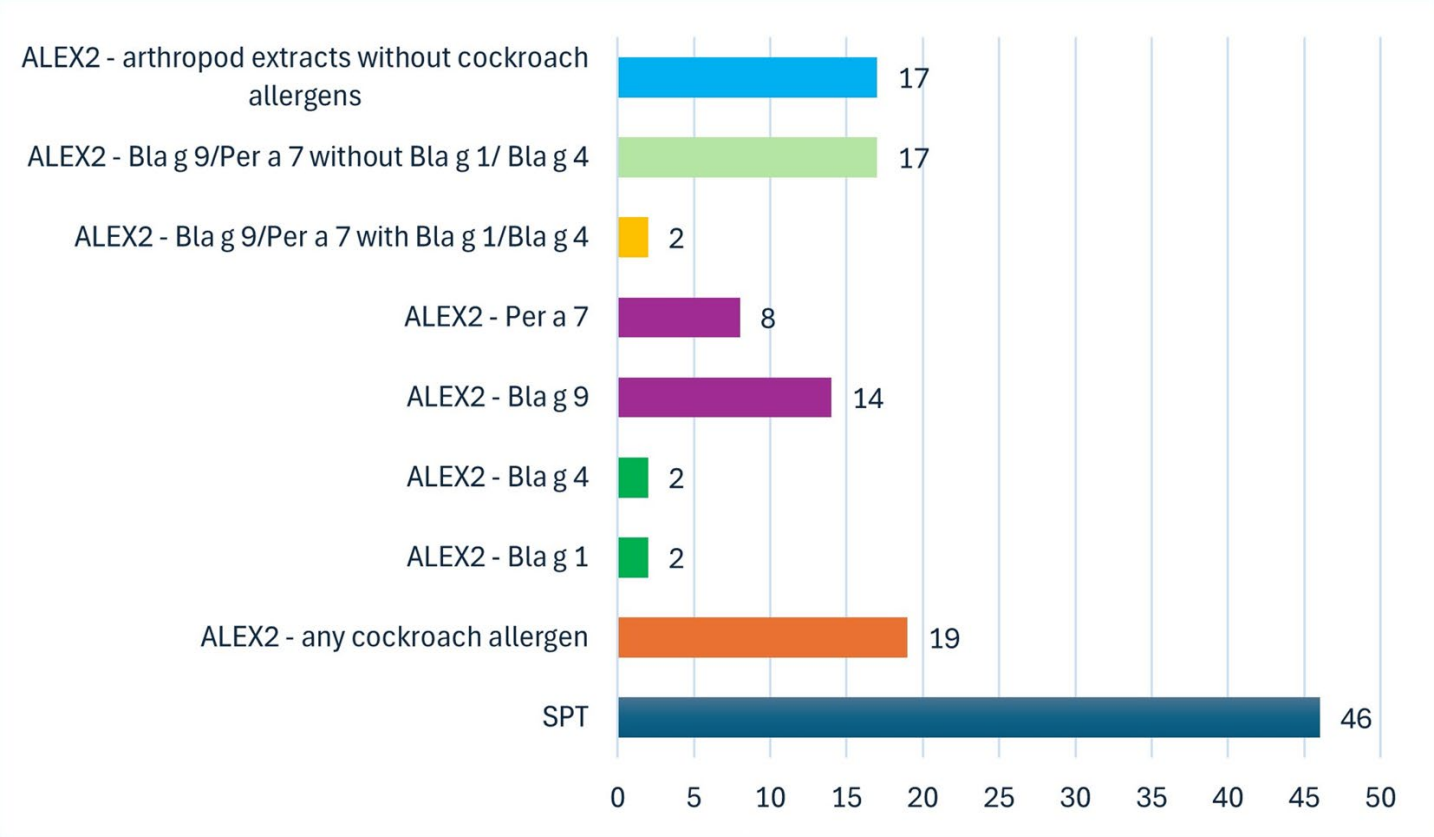
The number of participants with a positive SPT result for cockroach extract as well as a positive ALEX2 test results. Analysis was conducted only on individuals who had results for both tests (*n* = 46). SPT - skin prick test.

Based on data from individuals who have SPT and ALEX2 results (*n* = 46), we compared the positive results of these tests. In the ALEX2 test, a result of ≥ 0.3 kU/L was considered positive. An individual was counted as having a positive ALEX2 result if they had a positive result for at least one cockroach allergen. Our analysis showed that the number of people with a positive SPT does not reflect the number of people with a positive ALEX2 result (Fig. 2). Moreover, only 2 individuals had elevated IgE levels for both cockroach-specific allergens (Bla g 1 and Bla g 4), but no positive results for Bla g 2. Fourteen and eight patients were sensitized to the cross-reactive allergens Bla g 9 and Per a 7, respectively. Of these patients, two were sensitized to both cross-reactive allergens. Two patients who were sensitized to cross-reactive allergens were also allergic to at least one cockroach-specific allergen. One of them was sensitized only to Bla g 9 and both specific allergens, while the other one was sensitized to both cross-reactivity and both specific allergens. On the other hand, 17 patients were sensitized to cross-reactive allergens with no sensitization to cockroach specific allergens. Moreover, other 17 patients were neither sensitized to cockroach specific nor cross-reactive allergens but reacted with arthropod extracts.

### Analysis of sensitization based on the results of ALEX2 test

**Fig. 3:**
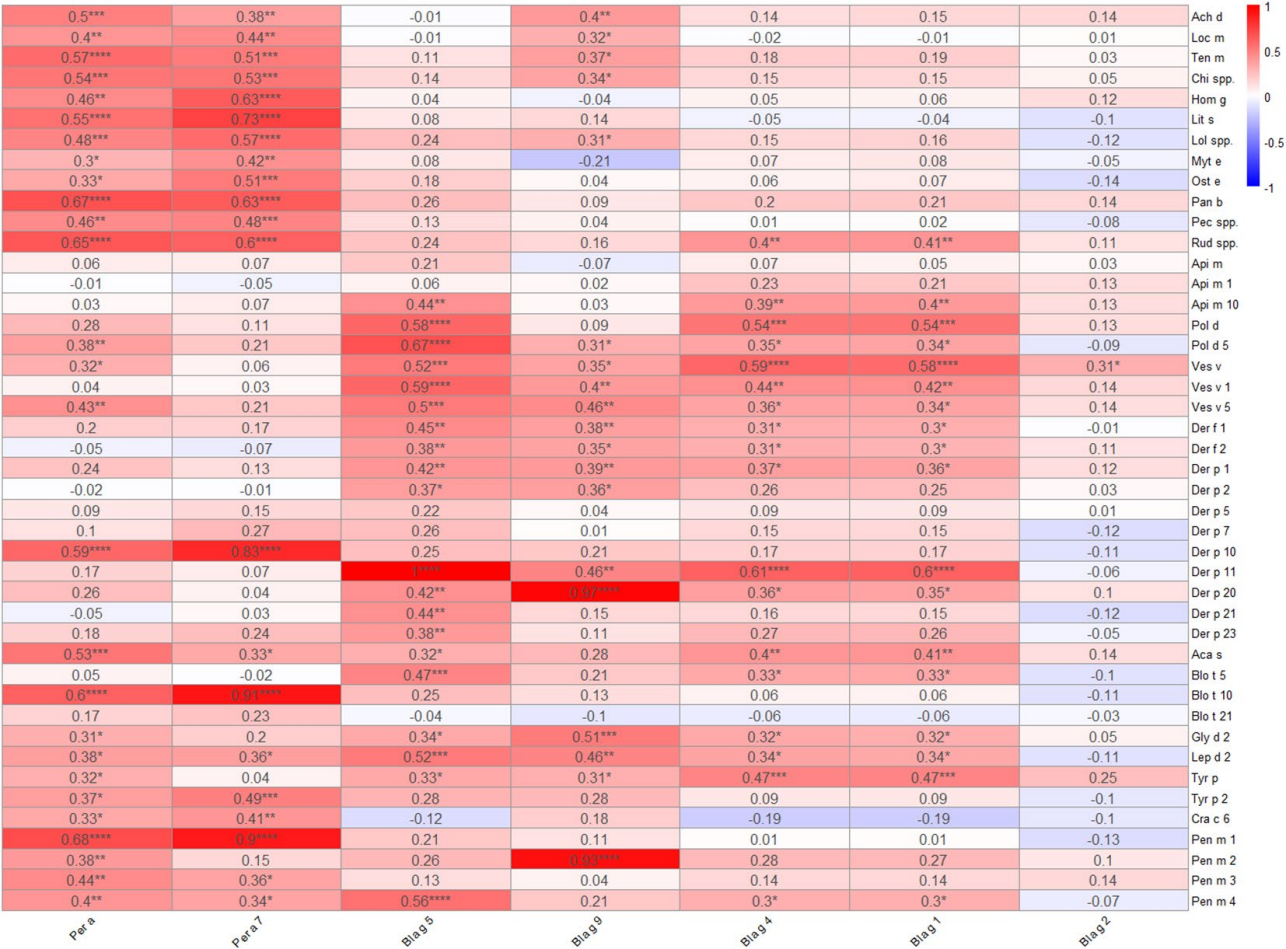
Correlation analysis of sensitization based on the results of ALEX2 test. Analysis conducted exclusively on data from individuals who obtained ALEX2 test results (*n* = 46). The values shown on the heat map are Spearman’s correlation coefficients. *p < 0.05, **p < 0.01, ***p < 0.001, ****p < 0.0001.

To further understand possible role of cross-reactive allergens in sensitization to cockroach extract, we conducted an analysis of the correlation between cockroach allergens, such as *Blattella germanica* (Bla g 1, Bla g 2, Bla g 4, Bla g 5, Bla g 9), *Periplaneta americana* (Per a, Per a 7) and selected allergens described in the methods section (Fig. 3).

First, we detected very strong positive correlations among cross-reactive allergens, such as Per a 7 and Bla g 9: Per a 7 and Der p 10 (rho = 0.82, *p* < 0.0001), Per a 7 and Blo t 10 (rho = 0.91, *p* < 0.0001), Per a 7 and Pen m 1 (rho = 0.9, *p* < 0.0001), Bla g 9 and Der p 20 (rho = 0.82, *p* < 0.0001), Bla g 9 and Pen m 2 (rho = 0.82, *p* < 0.0001), as well as Bla g 5 and Der p 11 (rho = 1, *p* < 0.0001). Interestingly, there are differences among specific allergens. Bla g 2 showed a significant correlation exclusively with Ves v. In contrast, the specific allergens Bla g 1 and Bla g 4 have similar correlation profiles – a strong correlation with allergen Der p 11, as well as Pol d and Ves v extracts. Secondly, our analysis shows correlations based on the species of cockroach. For example, Per a 7 from *Periplaneta americana* was significantly correlated with edible insects, such as cricket, locust, and mealworm; seafood; and storage mite. Similar, Bla g 9 was significantly correlated with edible insects, such as cricket, locust, and mealworm; some seafood and storage mite. However, unlike Per a 7, which only correlates with Der p 10, Bla g 9 correlates with most of the tested house dust allergens. Moreover, extract Per a was significantly correlated with extract Tyr p, as well as with Gly d 2 and Pen m 2 in contrast to cross-reactivity allergen Per a 7. Furthermore, there is a noticeable correlation with wasp species (*Polistes dominula* and *Vespula vulgaris*), unlike Per a 7.

## Discussion

Our study revealed cross-reactions among Polish PAR patients sensitized to cockroaches. Only a minor fraction of PAR patients with positive skin prick test results to *Blatella germanica* had elevated level of serum IgE to any of the cockroach-specific allergens. Most patients were characterized by elevated level of IgE to cross-reacting molecules, such as tropomyosins or arginine kinases. We observed very strong positive correlations between cross-reactive allergens, including those between Per a 7 and tropomyosins from house dust mites, storage mites and shrimp. This finding is consistent with previous study^13^. Cross-reactions resulting from IgE responses to common allergens, especially tropomyosin, may be caused by shrimps, mites, cockroaches, and *Ascaris lumbricoides*. Similar IgE-binding epitopes have been identified in tropomyosins from different invertebrates, which suggests that these proteins’ similar structure underlies the observed cross-reactivity^14^. Tropomyosin has been identified as the major allergen in shrimp, crab, lobster, dust mites, oyster, gastropod as well as cockroaches. Furthermore, tropomyosin from *Periplaneta americana* (Per a 7) demonstrates substantial sequence homology with invertebrate tropomyosins, especially those of dust mites and shrimp, with an identity of about 80%^15^. In Southern China, patients with shrimp allergy also exhibited co-sensitization to house dust mites (*Dermatophagoides pteronyssinus)*, cockroaches, crabs and moths. Those with allergic skin diseases had higher sIgE levels than patients with respiratory allergic conditions. Among other things, a strong correlation was detected between shrimp and cockroaches, crab and moth based on the optimal scaling analysis^16^. Similarly, a significant correlation was found between shrimp, dust mites, and cockroaches among children in both rural^17^ and inner-city areas^18^. However, cockroach allergens showed minimal cross-reactivity with seafood allergens but considerable cross-reactivity with house dust mite allergens in patients with AR^19^. Moreover, IgE cross-inhibition testing demonstrated that sensitization to house dust mites *(Dermatophagoides pteronyssinus)* may lead to false-positive skin prick test results for cockroach allergens in individuals with asthma and/or AR^20^.

Moreover, in a substantial part of our population, we were able to demonstrate elevated level of IgE to extracts of other arthropods yet no reaction to cross-reacting allergens including in ALEX2 test. Overall, we demonstrated a correlation between cockroach allergy and allergy to edible insects, such as cricket, locust, and mealworm; seafood; house dust mites and storage mites, as well as wasp species. This suggests that some other cross-reacting molecules may be present in arthropod extracts leading to positive skin prick test results seen in our patients. New cockroach molecules with allergenic potential have been described recently^21^. However, clinical manifestation of allergy is tightly dependent on the contact to allergens a patient is sensitized to^22^.

Exposure to indoor allergens, such as house dust mites, cats, dogs, rodents, and cockroaches, is widely associated with allergic rhinitis and asthma morbidity. Moreover, house dust mites and cockroaches are most commonly found in settled dust^23^. Exposure to cockroach allergens in the first quarter of life correlates with frequent episodes of wheezing and the development of asthma^15^. In Southeast Asia, the main allergens associated with allergic rhinitis are house dust mites, cockroaches, pollen, and mold^24^. Interestingly, hygiene practices during the pandemic did not lead to an increase in allergies to house dust mites *(Dermatophagoides farinae* and *Dermatophagoides pteronyssinus)*. However, an increase in allergies to German cockroaches, cats, and dogs was observed^25^. Furthermore, the prevalence of sensitization to house dust mites and American cockroaches was highest among children under 5 years of age with asthma and allergic rhinitis compared to children over 10 years of age with these conditions^26^. Curiously, in our study we detected a high correlation between Bla g 5 (glutathione S-transferase) and Der p 11 (paramyosin). According to sequence analysis, Der p 11 shows high homology to paramyosins derived from mites, ticks, and other invertebrates; therefore, sensitization to one of those proteins can be responsible for cross-reactivity between cockroach and dust mite extracts^27^. This less obvious cross-reaction with Bla g 5 may likely result from shared conformational epitopes of IgE or structurally similar domains. Although in a group of cockroach allergic patients in USA sensitization to Bla g 5 was relatively frequent no cross-reactivity with Der p 8 could be detected^28^.

Allergen-specific immunotherapy (ASIT) is used in the treatment of common allergic conditions, in particular allergic rhinitis, hypersensitivity to insect stings and allergic asthma^29^. Unfortunately, the number of patients with polysensitization has been growing recently. It is estimated that over 80% of patients with AR experience polysensitization. These patients have more severe clinical symptoms and a poorer quality of life. There are conflicting views in the literature regarding the efficacy of ASIT in patients with polysensitization^30^. However, our findings on sensitization patterns provide additional guidance for clinical decision-making by identifying the specific allergens to which patients are truly sensitized. Our study indicates that diagnosis of cockroach allergy based solely on extract-based testing may be, at least in some geographic regions, overestimated. Moreover, in vitro diagnostics which apply whole cockroach extracts is a subject to false positive results due to possible cross-reacting carbohydrate determinants present in the natural cockroach proteins^31^. Component-resolved diagnostics play an important role in making precise diagnoses, especially when identifying true and cross-reactive allergens. This approach ensures the correct allergens are selected for ASIT. Analyzing cross-reactive allergens provides important information about potential sources of sensitization and the associated clinical manifestations. While some of these allergens may cause severe symptoms, contact with others causes no symptoms^30^.

This study has several limitations. First, the analysis was based on a limited number of samples, which may reduce statistical power. The results may not be fully representative of the general population. Second, the study was conducted on the Polish population, which may limit the generalizability of the results to other populations. In addition, certain biological or environmental factors (e.g., medications, infections, allergen exposure) may have influenced the results, which were not controlled for in the study and may be a potential source of bias, including selection bias and confounding factors, such as age, sex and comorbidities. Furthermore, genetic predisposition (e.g., family history of atopy) and socioeconomic status (e.g., housing conditions, access to healthcare) may also contribute to the observed variability in allergic sensitization. These additions clarify the context of our findings and highlight the complexity of factors influencing allergic reactions. In summary, the results of the study indicate significant correlations between the analyzed variables, but they should be interpreted with caution and treated as a starting point for further, more controlled analyses.

In concluding, allergy to cockroaches correlates with sensitivity to edible insects, seafood, house dust mites, storage mites, and wasps. The pattern of significant correlations varies depending on the cockroach species, suggesting species-specific cross-reactivity. These findings advance our understanding of potential cross-allergens and may aid in allergy risk assessment and management in patients with AR.

## Methods

### Study design

It is part of a large study that analyzed the spectrum of allergies to specific allergens in patients with PAR. Among 250 consecutive patients with PAR (mean age 31.7 ± 14.1 years; 158 male and 92 female) living in the city of Bialystok and referred to the allergy clinic who were skin tested with a panel of aeroallergens, 48 (19.2%) were positive with *Blatella germanica* extract. Because the rate of positive test results with *Blatella germanica* was relatively high we decided to investigate the molecular basis of this sensitization. In addition to skin tests for other inhalant allergens, such as *Dermatophagoides pteronyssinus*, *Dermatophagoides farinae*, birch, grass, mugwort, cat, dog, *Alternaria*, an ALEX2 test was performed, and demographic data was collected from the participants.

All procedures used in the study were carried out in accordance with applicable guidelines and regulations, ensuring full compliance with ethical standards. All participants provided written informed consent. The study was approved by the Ethics Committee of the Medical University of Bialystok (approval number R-I-002/161/2016).

### ALEX2 test

The Allergy Xplorer (ALEX²) is an ELISA-based in vitro diagnostic assay designed for the quantitative determination of allergen-specific IgE. The ALEX² test can detect sIgE against 295 allergens simultaneously. These allergens come from 117 extracts and 178 molecular components from various sources, including foods, animals, plants, and molds. According to the manufacturer’s instructions, sIgE concentrations ≥ 0.3 kU/L were considered positive.

Particular extracts and allergens were selected for testing, such as *Blattella germanica* (Bla g 1, Bla g 2, Bla g 4, Bla g 5, Bla g 9), *Periplaneta americana* (Per a, Per a 7), *Acheta domesticus* (Ach d), *Locusta migratoria* (Loc m), *Tenebrio molitor* (Ten m), crab (Chi spp.), lobster (Hom g), shrimp (Lit s), squid (Lol spp.), mussel (Myt e), oyster (Ost e), shrimp (Pan b), scallop (Pec spp.), clam (Rud spp.), *Apis mellifera* (Api m, Api m 1, Api m 10), *Polistes dominula* (Pol d, Pol d 5), *Vespula vulgaris* (Ves v, Ves v 1, Ves v 5), *Dermatophagoides farina* (Der f 1, Der f 2), *Dermatophagoides pteronyssinus* (Der p 1, Der p 2, Der p 5, Der p 7, Der p 10, Der p 11, Der p 20, Der p 21, Der p 23), *Acarus siro* (Aca s), *Blomia tropicalis* (Blo t 5, Blo t 10, Blo t 21), *Glycyphagus domesticus* (Gly d 2), *Lepidoglyphus destructor* (Lep d 2), *Tyrophagus putrescentiae* (Tyr p, Tyr p 2), *Crangon crangon* (Cra c 6), *Penaeus monodon* (Pen m 1, Pen m 2, Pen m 3, Pen m 4).

### Statistical analysis

Statistical analyses were performed using R (version 4.2.2). Results with *p* < 0.05 were considered statistically significant. The Shapiro–Wilk test was used to check the data distribution. Spearman’s rank correlation test was used for correlation analysis. Statistical analysis of ALEX 2 test results was performed on raw sIgE concentrations obtained from the ALEX2 test, without dichotomizing the results. Raw values were treated as continuous variables reflecting the IgE response profile.

## Data Availability

All data generated or analyzed during this study are included in this published article.

## References

[1] Sharma, E. & Vitte, J. A systematic review of allergen cross-reactivity: translating basic concepts into clinical relevance. *J. Allergy Clin. Immunol. Glob.* **3**, 100230 (2024). 38524786 10.1016/j.jacig.2024.100230 PMC10959674

[2] García, B. & Lizaso, M. Cross-reactivity syndromes in food allergy. *J. Investig. Allergol. Clin. Immunol.* **21**, 2563 (2011). 21548443

[3] Pauli, G. Evolution in the understanding of cross-reactivities of respiratory allergens: the role of recombinant allergens. *Int. Arch. Allergy Immunol.* **123**, 183–195 (2000). 11112854 10.1159/000024443

[4] Arsenis, C., Taka, S. & Skevaki, C. Fundamentals of laboratory diagnostics in allergology. *Allergo J. Int.* **34**, 21–30 (2025).

[5] WHO/IUIS allergen nomenclature home page. https://www.allergen.org/index.php (2025).

[6] Matricardi, P. M. et al. EAACI molecular allergology user’s guide. *Pediatr. Allergy Immunol.* **27**, 1–250 (2016). 27288833 10.1111/pai.12563

[7] Dramburg, S. et al. EAACI molecular allergology user’s guide 2.0. *Pediatr. Allergy Immunol.* **34**, e13854 (2023). 37186333 10.1111/pai.13854

[8] Panzner, P. et al. Cross-sectional study on sensitization to mite and cockroach allergen components in allergy patients in the Central European region. *Clin. Transl. Allergy* **8**, 19 (2018). 29881542 10.1186/s13601-018-0207-x PMC5985581

[9] Mueller, G. A. et al. Characterization of an anti-Bla g 1 scFv: epitope mapping and cross-reactivity. *Mol. Immunol.* **59**, 200–207 (2014). 24667070 10.1016/j.molimm.2014.02.003 PMC4097036

[10] Stelmach, I. et al. Cockroach allergy and exposure to cockroach allergen in Polish children with asthma. *Allergy* **57**, 701–705 (2002). 12121188 10.1034/j.1398-9995.2002.23561.x

[11] Srisuwatchari, W. et al. Association between skin prick test and serum specific immunoglobulin E to American cockroach allergens in allergic rhinitis patients. *Allergol. Immunopathol.* **48**, 170–174 (2020). 10.1016/j.aller.2019.07.007 31601502

[12] Ayuso, R., Reese, G., Leong-Kee, S., Plante, M. & Lehrer, S. B. Molecular basis of arthropod cross-reactivity: IgE-binding cross-reactive epitopes of shrimp, house dust mite and cockroach tropomyosins. *Int. Arch. Allergy Immunol.* **129**, 38–48 (2002). 12372997 10.1159/000065172

[13] Mittermann, I. et al. IgE reactivity patterns in Asian and central European cockroach-sensitized patients reveal differences in primary sensitizing allergen sources. *J. Allergy Clin. Immunol. Glob*. **1**, 145–153 (2022). 37781268 10.1016/j.jacig.2022.04.003 PMC10509942

[14] Martins, T. et al. Reactions to Shrimp including severe anaphylaxis in mite- and cockroach-allergic patients who have never eaten shrimp: clinical significance of IgE Cross-reactivity to tropomyosins from different sources. *J. Investig. Allergol. Clin. Immunol.* **29**, 302–305 (2019). 31478527 10.18176/jiaci.0378

[15] Arruda, L. K. et al. Cockroach allergens and asthma. *J. Allergy Clin. Immunol.* **107**, 419–428 (2001). 11240940 10.1067/mai.2001.112854

[16] Liao, C. et al. Shrimp and cockroach co-sensitization in Southern China: association with moth sensitization. *Allergy Asthma Proc.* **41**, e54–e60 (2020). 32375970 10.2500/aap.2020.41.200013

[17] Yang, Z. et al. Cockroach is a major cross-reactive allergen source in shrimp-sensitized rural children in southern China. *Allergy* **73**, 585–592 (2018). 29072879 10.1111/all.13341

[18] Wang, J., Calatroni, A., Visness, C. M. & Sampson, H. A. Correlation of specific IgE to shrimp with cockroach and dust mite exposure and sensitization in an inner-city population. *J. Allergy Clin. Immunol.* **128**, 834–837 (2011). 21872304 10.1016/j.jaci.2011.07.045 PMC3185202

[19] Mohamad, S., Dam, E., Nurul, V. S. K., Chew, A. A., Abdullah, B. & F. T. & Cross-reactive IgE responses to cockroach, house dust mite, and seafood allergens in patients with allergic rhinitis. *Expert Rev. Clin. Immunol.* **21**, 1307–1314 (2025). 40911301 10.1080/1744666X.2025.2558060

[20] Sun, B. Q., Lai, X. X., Gjesing, B., Spangfort, M. D. & Zhong, N. S. Prevalence of sensitivity to cockroach allergens and IgE cross-reactivity between cockroach and house dust mite allergens in Chinese patients with allergic rhinitis and asthma. *Chin. Med. J. (Engl)*. **123**, 3540–3544 (2010). 22166627

[21] Wang, L. et al. Genome assembly and annotation of periplaneta americana reveal a comprehensive cockroach allergen profile. *Allergy* **78**, 1088–1103 (2023). 36153808 10.1111/all.15531

[22] Bégin, P., Waserman, S., Protudjer, J. L. P., Jeimy, S. & Watson, W. Immunoglobulin E (IgE)-mediated food allergy. *Allergy Asthma Clin. Immunol.* **20**, 75 (2024). 39736801 10.1186/s13223-024-00930-7 PMC11684040

[23] Beheshti, R., Grant, T. L. & Wood, R. A. Minimizing indoor allergen exposure: what works? *Curr. Allergy Asthma Rep.* **25**, 3 (2024). 39535667 10.1007/s11882-024-01185-3 PMC12379700

[24] Pham, D. L., Trinh, T. H. K., Le, K. & Pawankar, R. Characteristics of allergen profile, sensitization patterns and allergic rhinitis in SouthEast Asia. *Curr. Opin. Allergy Clin. Immunol.* **22**, 137 (2022). 35152227 10.1097/ACI.0000000000000814

[25] Zeng, Q., Yang, C., Li, J., Qiu, X. & Liu, W. Aeroallergen sensitization patterns and related factors in children with allergic rhinitis in Guangzhou. *Mediators Inflamm.* **2025**, 5887915 (2025). 10.1155/mi/5887915 PMC11879600 40041433

[26] Zahraldin, K., Chandra, P., Tuffaha, A. & Ehlayel, M. Sensitization to common allergens among children with asthma and allergic rhinitis in Qatar. *J. Asthma Allergy*. **14**, 287–292 (2021). 33824594 10.2147/JAA.S295228 PMC8018446

[27] Banerjee, S. et al. Der p 11 is a major allergen for house dust mite-allergic patients suffering from atopic dermatitis. *J. Invest. Dermatol.* **135**, 102–109 (2015). 24999597 10.1038/jid.2014.271 PMC4636057

[28] Mueller, G. A. et al. Analysis of glutathione S-transferase allergen cross-reactivity in a North American population: relevance for molecular diagnosis. *J. Allergy Clin. Immunol.* **136**, 1369–1377 (2015). 25930195 10.1016/j.jaci.2015.03.015 PMC4624055

[29] Moote, W., Kim, H. & Ellis, A. K. Allergen-specific immunotherapy. *Allergy Asthma Clin. Immunol.* **14**, 53 (2018). 30275845 10.1186/s13223-018-0282-5 PMC6157282

[30] Izmailovich, M. et al. Molecular aspects of allergen-specific immunotherapy in patients with seasonal allergic rhinitis. *Cells* **12**, 383 (2023). 36766723 10.3390/cells12030383 PMC9913438

[31] Holzweber, F. et al. Inhibition of IgE binding to cross-reactive carbohydrate determinants enhances diagnostic selectivity. *Allergy* **68**, 1269–1277 (2013). 24107260 10.1111/all.12229 PMC4223978

